# Assessment of the rabies education among middle secondary school students of southeastern Bhutan

**DOI:** 10.1371/journal.pone.0276862

**Published:** 2022-12-12

**Authors:** Lungten Lungten, Tenzin Tenzin, Sangay Rinchen, Kinzang Chedup, Sonam Wangchuk, Waraphon Phimpraphai, Michel de Garine-Wichatitsky

**Affiliations:** 1 City Veterinary Hospital and Satellite Laboratory, Dewathang, Samdrup Jongkhar, Bhutan; 2 Kasetsart University, Thailand and National Polytechnique Institute of Toulouse, Toulouse, France; 3 World Organization for Animal Health, Gaborone, Botswana; 4 National Centre of Animal Health, Department of Livestock, Thimphu, Bhutan; 5 District Veterinary Hospital, Samdrup Jongkhar, Bhutan; 6 Faculty of Veterinary Medicine, Kasetsart University, Bangkok, Thailand; 7 ASTRE, University Montpellier, CIRAD, INRAE, 34000, Montpellier, France; Zagazig University Faculty of Human Medicine, EGYPT

## Abstract

Rabies is one of the most important zoonotic diseases that mostly affect children. We conducted a rabies education among 129 secondary school children (intervention group = 94 students, control group = 35 students) in two schools in southeast Bhutan and evaluated the effectiveness of the lesson by comparing the knowledge, perception and safety behaviour score about rabies before and after education. We also assessed the knowledge retention capacity of the students at three months post intervention. Our findings indicated that short rabies lesson significantly (P<0.001) improved the mean knowledge score from 19.98(±2.72) to 26.96(±2.24) in the intervention group. Similarly, mean scores for perception and safety behaviour improved significantly (P<0.001) from 10.77 (±1.89) to 13.95 (±1.36) and 9.809 (±1.85) to 12.97 (±1.54), respectively. Although the scores have reduced significantly (P<0.001) at three months post intervention, most of the rabies information was largely retained by the students. In control group, significant increase in mean scores were also observed for perception from 10.17 (±2.38) to 11.2 (±2.44) and safety behaviour from 9.14(±1.44) to 10.74 (±1.95) after 3 months of education. The finding demonstrate that a short rabies lesson is effective in improving knowledge, perceptions and understanding of dog bites safety behaviour among the school children. However, there is a need for a frequent awareness program, at least quarterly or half yearly. Rabies education should focus on critical points such as dog bites being the main source of rabies and the importance washing a dog/animal bite wound with soap and water, and visiting the hospital for medical advice following animal bites.

## Introduction

Rabies is a fatal zoonotic disease that kills around 59,000 people every year all around the world with the majority of cases being reported from Asia and Africa [1,2]. The disease is mainly transmitted through dog bites [2]. It is estimated that three billion people in the world are at risk of getting rabies infection with 1.4 billion from south-east Asia [1,3]. Although rabies is 100% fatal after showing clinical signs, it is also a 100% preventable disease by vaccinating dogs, avoiding dog bites, immediate washing of bite wounds with soap and water, and timely administering post-exposure prophylaxis [2].

Due to their smaller stature, low level of awareness and high curiosity towards dogs, children are reported as common victims of dog bites and rabies[4,5]. Children might not be able to recognize emotions and behavioral signals of the dogs and often confuse an angry dog and a friendly dog thereby resulting in bites [6]. Sometimes children out-rightly provoke dogs by throwing stones and other objects when they see them on the streets [7]. In other times, due to their love for dogs they often play with the dogs [8]. Further, due to the fear of scolding, children would hide and do not report bite incidents to their parents [9]. In some instances, rather than seeking medical care, parents prefer to seek traditional treatments for bite wound [7,10]. Therefore, since children are at high risk of getting bitten by dogs and subsequent by rabies virus infection, it is extremely important to educate children on dog bites and rabies.

School-based rabies education is considered as one of the most efficient methods of reaching large number of children [11]. There are numerous school-based education tools which have proved to be effective for educating children [12]. The integration of rabies information into school curriculum in the Philippines and Sri Lanka (Rabies Edutainment 4 Kids) resulted in a significant improvement in rabies awareness levels and reduction of bites incidents in students [13,14]. Audio-visual lessons [10] and education using graphic images and videos [15] in secondary schools of India improved students’ knowledge related to local treatments of rabies and knowledge related to interpretation of dog behaviours respectively. Teaching primary school students on judging the behaviour of the dogs using a workbook, video and role-playing with life-size toy dogs in the ‘BARK (Be Aware, Responsible, and Kind)’ education program significantly increased the proportion of correct answers with more number of students able to identify danger level of a dog on basis of dog’s body language following the lesson [16]. Behavioural observations, seven to ten days after training by dog handlers using real dogs on how to identify dog states (e.g. friendly, angry, frightened) and how to behave safely around dogs, (e.g., how to approach dogs) indicated that children who had received the intervention displayed greater precautionary behaviour than children in the control schools (who had not received any intervention [17].

In Bhutan, rabies is endemic in the southern parts of the country that shares a border with India [18]. Dogs bites is very common with a report of around 7000 dog bites (1026 bites per 100,000 people) incidents every year [19]. School children are most common dog bite victims particularly those who are between the age groups of 5–9 years [20]. Because of high incidences of dog bites in children, awareness education in schools are conducted every year, particularly on World Rabies Day– 28 September–in the form of rabies talk, radio talk, TV panel discussion, and airing of documentary video about rabies [21,22]. Power point presentations on rabies by animal and human health workers have been commonly given to school children. However, due to logistic constraints, the presentations cover only a limited number of schools with more focus in urban areas. Moreover, although the Power point presentations are provided by gathering large numbers of students, the education program may not be producing the expected change. Due to sheer numbers of students, it is possible that some of the students may not concentrate during the education program. Unfortunately, post education assessment has not been conducted to understand the effectiveness of such rabies awareness programs. Therefore, we conducted this study to assess the impact of rabies awareness education among school children in two middle secondary schools in south eastern Bhutan. The information generated from this study is expected to provide guidance in preparing an appropriate rabies prevention and control programs in school children in the country.

## Materials and methods

### Study area

**Fig 1:**
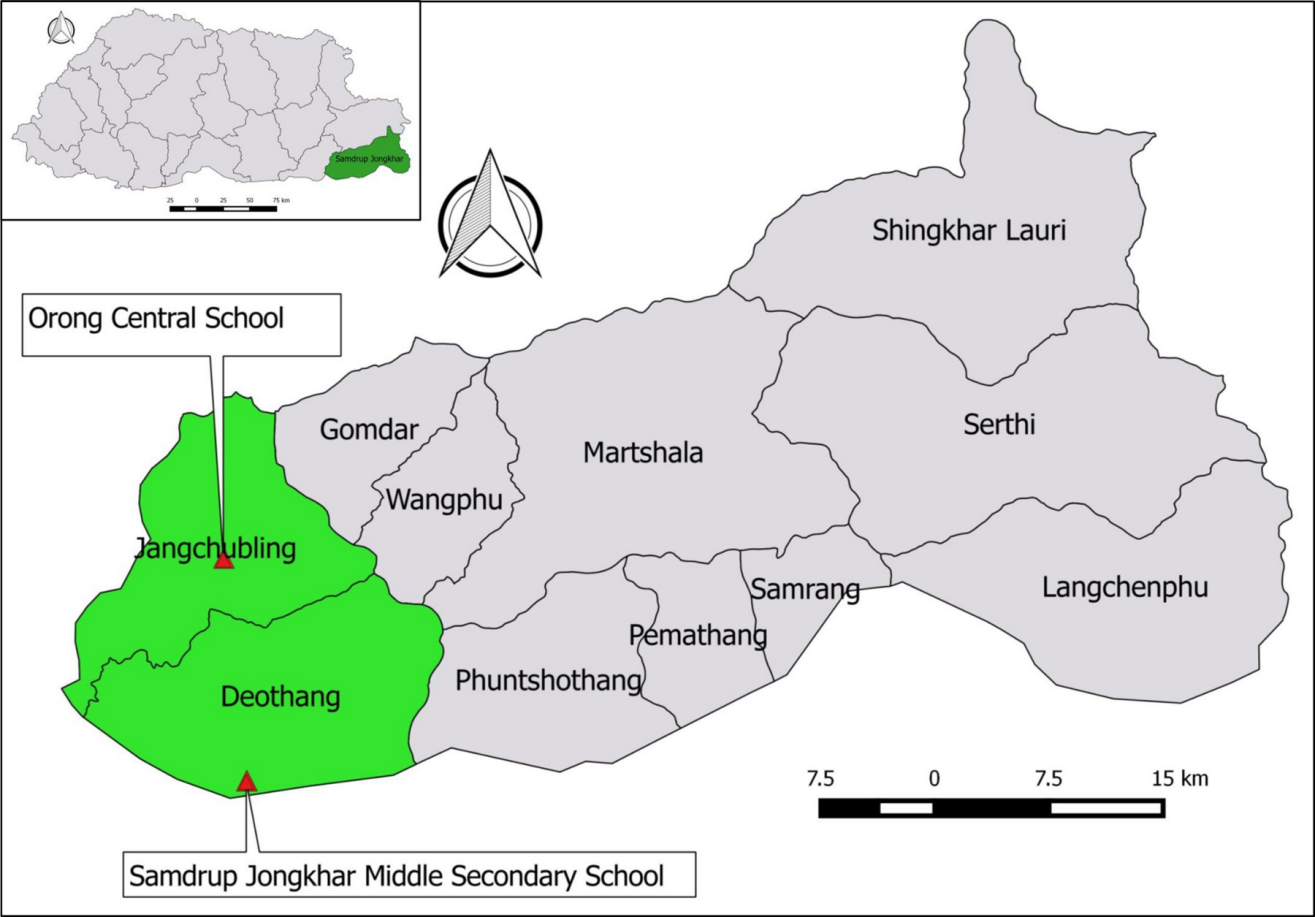
Map of Bhutan showing the location of two study schools—Orong Central School Samdrup Jongkhar Middle Secondary School) The maps were generated using QGIS software (QGIS Development Team, 2019).

The study was conducted in two schools—Orong Central School (OCS) and Samdrup Jongkhar Middle Secondary School (SJMSS) that are located under Samdrup Jongkhar district (SJ), south-eastern Bhutan (Fig 1). The district had a total of eight middle secondary schools. However, due to COVID-19 pandemic and lockdown, entry into some school campus were denied. Therefore, based on the risk assessment and frequency of rabies outbreaks, approvals were sought for only two selected schools. The SJMSS is located in the centre of the Samdrup Jongkhar town that shares a border with an Indian town of Darranga inAssam State where cross-border movement of the dogs are seen frequently. The OCS is located about 67 km north from Samdrup Jongkhar town in which sporadic cases of rabies outbreaks had been reported [23–25].

### Study design

We designed an educational interventional study to evaluate the effectiveness of rabies education in improving knowledge and prevention aspects of risky behaviours for dog bites among school children. In order to reduce potential biases related to age and different grades of the students surveyed, we enrolled only grade X students from both schools. All the students studying in grade X were incldued in the study. There were three sections in grade X, and one section from each school was randomly assigned to a control group and the rest as intervention group. Ninety four students (50 students from OCS and 44 students from SJMSS) were alloacted in the intervention group and 35 students in the control group (18 students from OCS and 17 students from SJMSS).

### Questionnaire design

A structured questionaire was designed, consisting of three sections: Section A focusing on socio-demographic information of the school children such as age, sex, and parents’ occupations; Section B containing information related to knoweldge regarding rabies such as the causes of rabies, suceptible hosts, mode and route of transmission, clinical signs and rabies preventive measures, students’ perceptions related to post expousre prophylaxis (PEP) and student’s likely actions after seeing a suspect rabid dog; Section C containing questions related to the understanding and recognition of dog behaviours and adoption of safety behaviours when interacting with dogs for prevention of dog bites. The questionaires were developed by the lead researcher (S1 File). However, some of the contents of the questionaires were adopted from similar studies conducted in other parts of the world [11,15]. The quesionaires were reviewed several times by supervisors and checked for reliability, clarity, relevance and appropriateness, and was pre-testedwith 10 students from SJMSS prior to actual survey. Necessary changes were made accordingly in the final survey questionaires.

#### Awareness education program

The students from the intervention group were gathered together in a multi-purpose hall and were provided a 45 minutes power point presentation on rabies (rabies lessons), while the control group were not provided any information on rabies. In order to ensure that educational message were not transferred to control groups, all students in the control groups were kept in the classrooms until the completion of powerpoint presention and assessment to the intervention groups. The educational presentation was given by the lead author of this paper. The presentation covered several aspects related to rabies, such as causes of rabies, rabies transmission routes, rabies symptoms, rabies preventive and first aids measures, and how to behave with dogs. The contents of the presentation was prepared by assembling relevant and important aspects on rabies, and the materails were sourced from the Global Alliance for Rabies Control (GARC) [26], World Health Organization (WHO) [27], and World Organisation for Animal Health websites [28]. The power point slide was evaluated for its contents and endorsed by supervisors of this study. At the end of the presentation, the students were invited to ask questions. The question-answers session lasted for around 30 minutes. The students were also allowed to ask any questions during the course of the presentation. The presentation was done in English and whenever necessary, it was also presented using Dzongkha (national language) or the Sharchop (native language of eastern Bhutan) for better understanding by the students. The rabies education program was conducted on 2^nd^ July, 2020 in Samdrup Jongkhar Middle Secondary School and on 3^rd^ July, 2020 in Orong Central Sschool.

### Data collection

**Fig 2:**
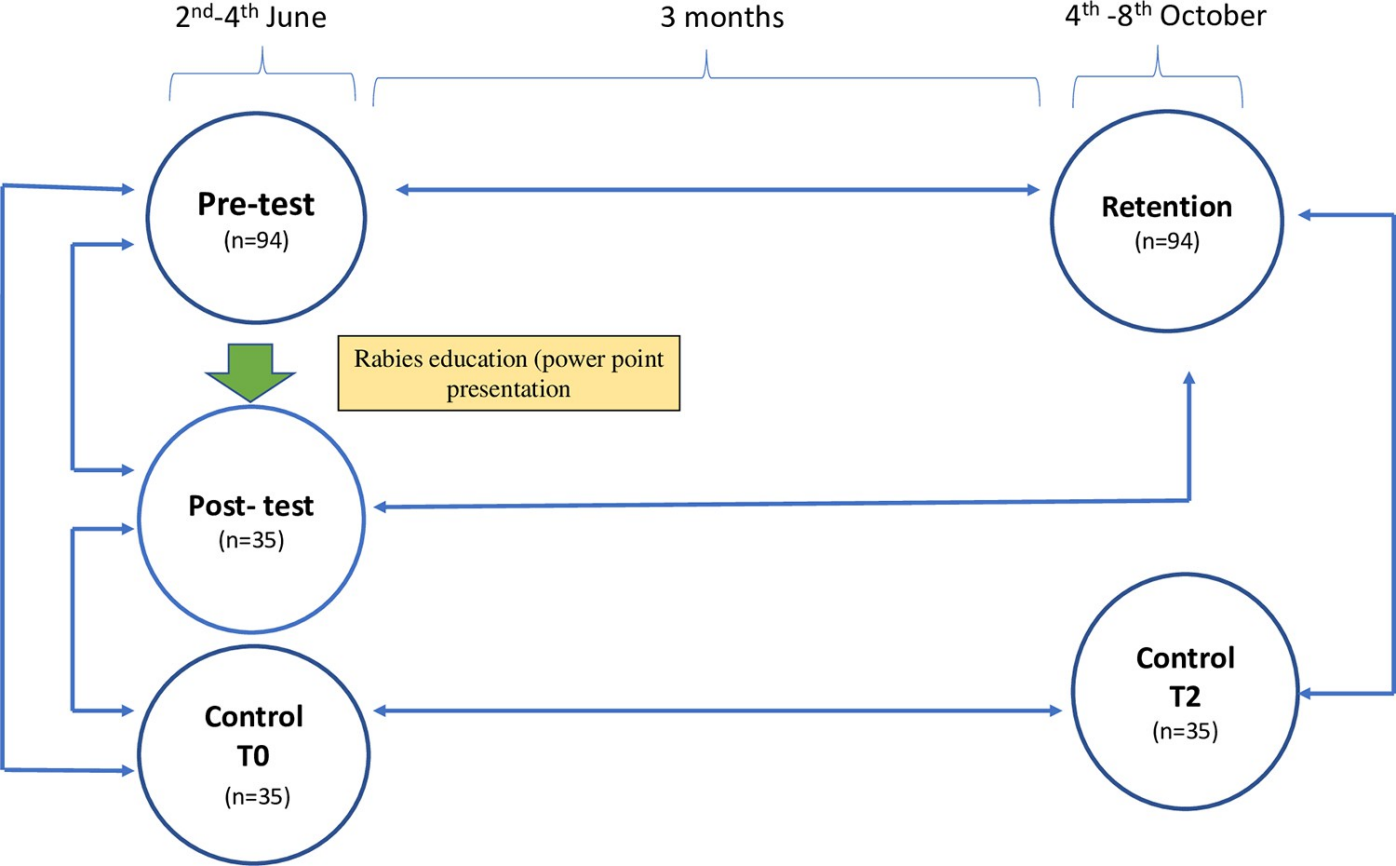
Schematic diagram of data collection, educational intervention and data analysis. The pre-test represents the data collection before educational intervention, post-test correspond to data collection immediately after intervention (after power point presentation on rabies) and retention correspond to the data collection at three months time period at after intervention. Control T0 and Control T2 refers to data collection from control group on day 0 (Control T0) and at three months time period (Control T2). The data collection from the control group was also done at the same time of intervention group. The numerical figures refer to the number of students participated in the survey in each study group at different time points.

The data was collected at three different time points in order to evaluate the variations of knowledge of the students: on the day of education program, i.,e before the intervention/education (T0) and considered as pre-test; immediately after the presentation/educational program (T1) and considred as post-test; three months after the educational intervention (T2) and considered as retention test. Knowledge, attitude and safety behaviour around dogs were assessed using the same questionnaire at all three time periods (pre-test, post-test and retention test) in the intervention groups, whereas in the control groups (the group that did not attend rabies education), data collection was done only two times during the pre-test (control T0) and after three months (control T2). The same quesionaire was used for both intervention and control groups, and also for all three time points. The questionnaires were distributed to the students and were self-administred by the students. The students were provided guidance on how to answers the question, in both intervention and control groups (pre-test, T0). The same questionnaire was self-administred by the intervention group students immediately after the rabies education power point presentation (post-test T1). Similarly, the two schools were re-visited after three months and collected the self-adminstered questionnaire from both the intervention and control groups (retention, T2). The overall framework of the intervention and data collection is presented in Fig 2. No interventions in any forms related to rabies were provided in both schools until the completion of this study.

Prior to their voluntary participation, the students were clearly explained about the research and education objectives of the study. A written informed consent to participate in this study was collected from each student and none of the students had refused participation. Administrative approvals were also obtained from the district education officers, and from the respective school principals and each class teacher involved prior to the study. The study was approved by the Research Ethics Board of Health (REBH), Ministry of Health, Royal Government of Bhutan vide letter no. Ref. No. REBH/Approval/2019/113. The study was conducted from 2 June to 8 October, 2020.

### Data management and analysis

The data were entered into a database developed in EpiInfo software version 7.2.3.1 [29]. The data were then exported into Microsoft excel 2013 (Microsoft Excel, Redmond, USA) and checked for any errors before performing analysis.

The answer for each question were examined and allocated a score points. The answers to the questions which were considered of critical importance that the students should have knowledge and understanding of it were allotted a score of “2” points. For example, it is important that students should know that rabies is a fatal disease, and that dogs are the main source of rabies, and that rabies is transmitted through dog bites. Other answers to questions which were in agreement with the current conventional medical knowledge about rabies, but which were not deemed so much important in terms of outcomes for the students, were allotted a score of “1” point. For example, transmission of rabies by domestic animals (livestock) is possible but is not reported frequently, and if the students answer livestock as the source of rabies, a score of “1” was allotted accordingly. A score of “0” was allocated to all wrong answers and to those answers where the respondent indicated that he/she was not sure about it (e.g., I don’t know). For example, if the students answered that snakes and birds are the main host of rabies, and rabies can be transmitted from food and feces, they were allotted a score of “0” point. The scoring system adopted to each question is shown in Table 1. If the students answered all the questions correctly, they would obtain a total score of 58 points. Similarly, if the students have answered all questions correctly the students could obtain a maximum of 29 points for rabies knowledge related questions, 14 points for perception questions and 15 points for safety behaviour questions (see Table 1).

**Table 1:** Socio-demographic characteristics of the students who participated in the rabies education interventional study from two schools in Samdrup Jongkhar District, South eastern Bhutan (n = 129).

		Groups		χ2
Characteristics	Total (n = 129)	Intervention (n = 94)	Control (n = 35)	p value
	n(%)	n(%)	n(%)	
**Schools**				
OCS^a^	68 (52.7)	50 (53.2)	18 (51.4)	0.858
SJMSS^b^	61(47.3)	44(46.8)	17(48.6)	
**Gender**				
Male	61(47.3)	57 (60.6)	4(11.4)	<0.001*
Female	68(52.7)	37(39.4)	31(88.6)	
**Age**				
Adolescent	55 (42.6)	47(50)	8(22.9)	0.006
Young	74(57.4%)	47(50%)	27(77.1%)	
**Fathers’ occupation**				
Self employed	75(58.1)	60 (63.8)	15(42.9)	0.032
Employed	54(41.9)	34(36.2)	20(57.1)	
**Mothers’ occupation**				
Self employed	113 (87.6)	82 (87.2)	31(88.6)	0.552*
Employed	16 (12.4)	12(12.8)	4(11.4)	
**Own dog**				
No	103(79.8)	70 (74.5)	33(94.3)	0.008*
Yes	26(20.2)	24(25.5)	2(5.7)	

^a^Orong Central School.

^b^Samdrup Jongkhar Middle Secondary School.

*Fisher’s Exact Test.

Data analysis was performed in R statistical software version 3.6.1 [30]. Descriptive analysis was performed by calculating the proportions, frequency, mean and standard deviation. Using the mean age of 16 years, the age was categorized as “adolescent” for those students whose age was more than 16 years and “young” for those whose age was less than or equal to 16. The occupation of the father and mother of the students were collapsed into two categories as “employed” for those working in military, government offices and corporate offices while those doing business, farming, and others activities were categorized as “self-employed”. Frequencies of the categorical variables related to socio-demographic characteristics and dog ownership status were compared between intervention and control group using Pearson’s Chi-squared test with yates’ continuity correction. When the frequencies were less than 5, comparisons were made using fisher’s exact test.

The overall scores, and scores for each domain of interest such as knowledge, perception and safety around dogs were calculated for each student of different groups. Data in the intervention groups were matched by collecting the data from same students during all three stages of data collection (pre-test, post-test and retention-test). Since the mean scores were not normal, comparison between pre-test and post-test were done using Wilcoxon signed rank test to assess the immediate impact of the rabies education program. The mean scores comparison between pre-test and post-test were also compared with control groups using Mann Whitney test (unmatched) to see how the scores differed between intervention and control groups. In order to evaluate how much knowledge is retained by the students up to three months, the mean scores of pre-tests and post-test were compared with the mean scores at three months (retention test) using Wilcoxon signed rank test. The mean scores of post-tests were also compared with mean score of control groups (Control T0) to see how intervention groups differ from control groups using Mann Whitney test. The mean scores of control group at day 0 (Control T0) was also compared the mean score of the same control group at three months (Control T2) using the Wilcoxon signed rank test. A logistic regression model was built on baseline scores to find out how socio-demographic variables were associated with the baseline knowledge on rabies among the students. The odd ratios, 95% confidence interval and p-values were calculated.

## Results

### Socio-demographic characteristics of the students

A total of 135 students participated in the survey but only 129 (95.6%) students (intervention group- 94, control group-35) completed the questionnaires and were included in the analysis. From the total of 129 students who completed the data, 52.7% (n = 68) were females and 47.3% (n = 61) were males. The mean age of the students was 16.4 years. The students from Orong Central School (52.7%, n = 68) were boarder and living in the school campus, while the students from Samdrup Jongkhar Middle Secondary School (47.3%, n = 61) were day-scholars. Table 1 describes the demographic details of the students.

### Baseline knowledge, perception and safety behaviour scores (pre-test result)

**Fig 3:**
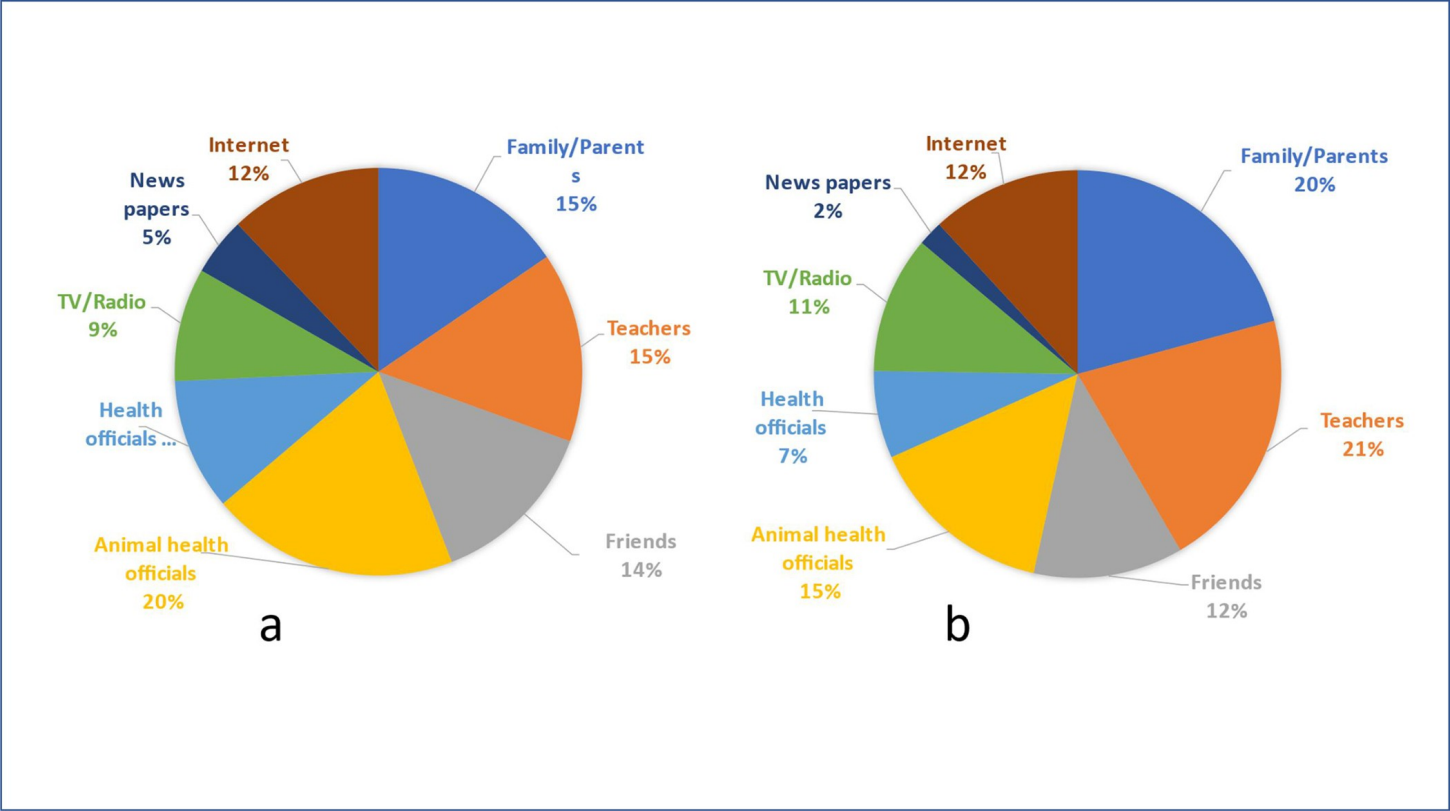
Source of information on rabies at day T0 (pre-test questionnaire survey) among students: a) Intervention group (n = 94), and b) Control group (n = 35).

All students (100%) in both the intervention and control groups had heard about rabies before the rabies education with power point presentation. Animal health workers, family, teachers and friends were cited as the most important source of rabies information by students in both intervention and control groups (Fig 3).

### Overall knowledge, perception and dog bite safety scores

The average overall knowledge score of the students in the intervention groups was 40.55 (±4.27), with all students able to correctly answers 69.9% of the questions. The average overall scores in the control group were 39.46 (±7.09) with 68.0% of the questions correctly answered by all students. There was no significant (Wilcoxon sum rank W = 1627.5, P = 0.928) difference in the mean scores between the intervention and control group before rabies education (Table 2).

**Table 2:** Table showing differences of scores of students between intervention group (students that receives rabies education) and control groups (students that did not receive rabies education).

Groups	Groups		
	Intervention	Control	P value
**Pretest**			
Overall score	40.55 (4.27)	39.46(7.09)	0.928
Knowledge score	19.98(2.72)	20.14 (5.06)	0.073
Perception score	10.77(1.89)	10.17 (2.38)	0.229
Safety score	9.809 (1.85)	9.143(1.44)	0.025
**Post test**			
Overall score	53.87(3.98)		
Knowledge score	26.96(2.24)		
Perception score	13.95(1.36)		
Safety score	12.97(1.54)		
**Retention**			
Overall score	47.68(5.25)	43.29(5.84)	<0.001
Knowledge score	23.05(2.80)	21.34(3.44)	0.015
Perception score	12.21(2.20)	11.2(2.44)	0.037
Safety score	12.41(1.64)	10.74(1.95)	<0.001

#### Knowledge score

The mean knowledge score in the intervention group was 19.98 (±2.72) and 20.14 (±5.06) in control group. This corresponds to 68.9% correct answers in the intervention group and 69.5% correct answers in control group. No significant differences were observed in the knowledge score between intervention and control groups (W = 1308.0, P = 0.073) (Table 2). In both the groups, majority of the students were able to correctly identify dogs as main source of rabies with 96.8% (n = 93) students from intervention groups and 94.3% (n = 33) students from control group. About 76% (n = 75) of the students from intervention and 77.1% (n = 27) from control groups knew that there is no treatment for rabies and that it is a fatal disease. The students in the intervention group correctly identified the transmission routes of rabies as dog bites (93.6%, n = 88), scratches (62.8%, n = 59), and licks (39.4%, n = 37), while in the control group they correctly identified transmission routes of rabies as dog bites (77.1%, n = 27), scratches (62.9%, n = 22) and via licks (51.4%, n = 18). Similarly, aggressiveness (76.6%, n = 72), salivation (77.7%, n = 73) and abnormal barking (56.4%, n = 53) were identified as main clinical signs of rabies by students in intervention group, while in the control group, they correctly mentioned the signs as aggressiveness (60.0%, n = 21), salivation (74.3%, n = 26) and abnormal barking (57.1%, n = 20). No significant differences were observed in the specific knowledge score between intervention and control groups (P>0.05). The details of the specific knowledge score of the students for both the intervention and control is presented in S2 Table.

#### Perception score

The mean perception score of the students in the intervention group was 10.77 (±1.89) and in control group the mean score was 10.17 (±2.38), which correspond to 76.9% and 72.6% of the correct answers, respectively. No significant (W = 1869.5, P = 0.229) differences were observed in the perception scores between intervention and control groups (Table 2). When students were asked what they would do if they were bitten by a rabid dog, immediate washing of the bite wound (85.1%, n = 80), reporting to parents or teachers (83.0%, n = 78) and visiting hospital for anti-rabies vaccination (97.9%, n = 92) was correctly mentioned by the students in the intervention group. In the control group immediate washing of the bite wound (74.3%, n = 26), reporting to parents or teachers (57.1%, n = 20) and visiting hospital for anti-rabies vaccination (91.4%, n = 32) were also correctly answered by most of the students. The favorable perceptions of reporting the suspected cases of rabies to both teachers and parents (60.6%, n = 57), livestock officials (81.9%, n = 77) and alerting nearby people (70.2%, n = 66) was also reported by most students in the intervention groups. However, in the control groups, only one-third of the students (34.3%, n = 12) mentioned that they would report cases of rabies to teachers and parents, but most students mentioned that they would report to livestock officials (62.9% (n = 22) and alert nearby people (54.3% (n = 19). The details of responses to individuals’ questions related to perceptions on rabies from both intervention and control groups is presented in S2 Table.

#### Dog bite safety measures scores

When assessing the dog bite prevention measures, the intervention group obtained a mean score of 9.81 (±1.85) which corresponded to 65.4% correct answers. In the control group, mean score was 9.143 (±1.44) which corresponds to 60.9% of the correct answers. There was a significant difference observed between two groups (W = 2061, P = 0.025). Most of the students from both intervention group (70.2%, n = 66) and control group (74.3%, n = 26) believed that it is safe to approach and provide food to dogs. Most students from the control group (60%, n = 21) mentioned that it was safe to intervene and separate dogs when they see them fighting in the street, while only 36.2% (n = 55) of the students from intervention group had the same opinion. The proposed behavior to be adopted when a dog is approaching towards a person for biting, “standing still without moving” (52.1%, n = 49) and “scrolling down by covering face or head with clothes” (48.9%, n = 46) were mentioned by half of the students in the intervention group, which is higher compared to the control groups (20% to 42%). Most students 77.7% (n = 73) in the intervention group mentioned that “running away” is the best options when a dog starts to bite, while 91.4% (n = 32) gave the same response from the control group. The detailed responses of the students related to the prevention of dogs’ bites is provided in S2 Table.

Logistic regression model showed that factors such as schools (SJMSS), mother’s occupations (employed) and age of the students (adolescent) were significantly associated with higher baseline knowledge on rabies among the students (Table 3).

**Table 3:** Final multivariable logistic regression model to determine factors associated with baseline knowledge (pre-test questionaries) on rabies among the students (n = 129).

Categories	Estimates	Std. Error	Adj. OR (95%CI)	p value
**Age**				
Young	Reference			
Adolescent	0.78	0.39	2.2(1.0–4.7)	0.047
**Name of schools**				
^a^OCS	Reference			
^b^ SJMSS	0.87	0.39	2.4(1.1–5.2)	0.027
**Mother’s occupation**				
Self employed	Reference			
Employed	2.33	1.07	10.3(1.9–193.2)	0.029

^a^Orong Central School.

^b^Samdrup Jongkhar Middle Secondary School.

### Immediate impact of the rabies education (post-test result)

#### Overall scores

**Fig 4:**
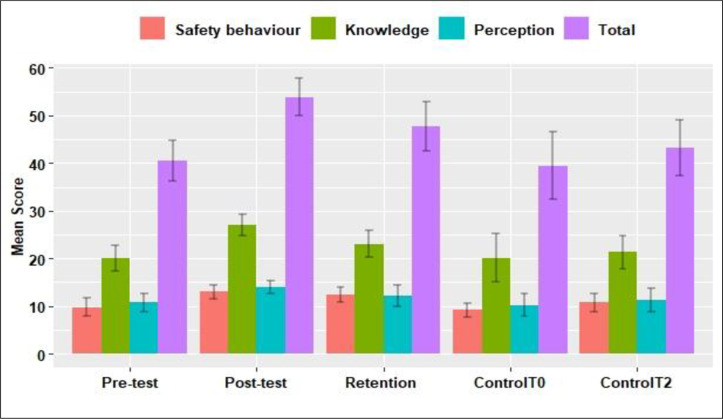
Bar graph showing the students mean scores (+/- Standard Errors) of knowledge, perception and safety behaviour around dogs at different time points.

After the educational lesson (i.e., power point presentation on rabies to the students in intervention group), the overall mean score significantly increased from 40.55 (±4.27) to 53.87(±3.98) which correspond to increase of correct answers from 68.7% before the rabies awareness program (pre-test) to 91.1% immediately after education on rabies (Mann-Whitney test 3.5, P<0.001) (Table 4 and Fig 4).

**Table 4:** Table showing the changes of scores of the students in intervention groups: Pre-test (before the rabies education), post-test (immediately after education) and retention (three months after rabies education).

Groups	Time period			Significant differences		
	Pre-test	Post-test	Retention test	p value (Pre vs Post)	p value (Pre vs Retention)	p value (Post vs Retention
**Intervention group**						
Overall score	40.55 (4.27)	53.87(3.98)	47.68(5.25)	<0.001	<0.001	<0.001
Knowledge score	19.98(2.72)	26.96(2.24)	23.05(2.80)	<0.001	<0.001	<0.001
Perception score	10.77(1.89)	13.95(1.36)	12.21(2.20)	<0.001	<0.001	<0.001
Safety score	9.809 (1.85)	12.97(1.54)	12.41(1.64)	<0.001	<0.001	0.023
**Control group**						
Overall score	39.46(7.09)	39.46(7.09)	43.29(5.84)		0.005	0.005
Knowledge score	20.14 (5.06)	20.14 (5.06)	21.34(3.44)		0.157	0.157
Perception score	10.17 (2.38)	10.17 (2.38)	11.2(2.44)		0.041	0.041
Safety score	9.143(1.44)	9.143(1.44)	10.74(1.95)		0.001	0.001

#### Knowledge scores

The mean knowledge scores significantly (V = 11.5, P<0.001) increased from 19.98 (±2.72) to 26.96(±2.24) after intervention, corresponding to an increase of correct answers from 68.90% before rabies awareness program (pre-test) to 92.97% correct answers after the intervention (Table 4). When the individual specific questions were assessed, there was a significant improvement in knowledge scores after the intervention education (S3 Table).

#### Perception scores

There were significant (V = 55, P<0.001) improvements of favorable attitude among students after the education related to caring of dog bites wound and their reporting (Table 4). The mean scores increased from 10.77 (±1.89) to 13.95 (±1.36) which corresponds to an increase of correct answers from 76.93% to 99.64%. Similarly, significant improvements in perceptions related questions were observed after education (S3 Table).

#### Dog bites safety behaviour

There were also significant (V = 62.5, P<0.001) improvements of students understanding of dog behaviors and bite prevention safety measures (Table 4). The mean scores increased from 9.809 (±1.85) to 12.97 (±1.54) that correspond to overall improvement of correct answers of the questions from 61.3% before the lesson to 81.1% after the lesson (Table 4). Looking at the impacts in individual questions related to safety behaviour after the lesson, there were significant improvements in scores of all questions except for one in which students feels it is safer to play with puppies than adult dogs (S3 Table).

When mean scores were compared between intervention and control groups, overall mean score, knowledge mean score, perception mean score and safety mean score in intervention group were significantly higher than the mean score of the control group (Table 2).

### Knowledge retention capacity at three months

#### Overall scores

When the mean scores after rabies lesson (post-test) and at three months (retention test) were compared, the overall mean scores significantly (V = 3336, P<0.001) reduced from 53.87(±3.98) to 47.68(± 5.25) at three months, which corresponds to a reduction of correct answers from 91.14% to 80.81% (Fig 4). However, the overall mean scores at three months were still significantly (V = 224, P<0.001) higher than the baseline scores (pre-test) when the students answered only 68.7% of the questions correctly (Table 4 and Fig 4).

In the control groups, the overall mean scores significantly increased (V = 122.5, P = 0.005) from 39.46(±7.09) at pre-test to 43.29 (±5.84) at three months, which corresponds to an increase of correct answers from 68.0% to 74.6% (Table 4). Significant differences (W = 935.5, P<0.001) in the overall mean scores were observed between intervention and control groups at three months period where students in the intervention group have answered higher proportion of correct answers compared to control group (Table 2).

#### Knowledge scores

The mean knowledge scores significantly (V = 3803.5, P<0.001) reduced from 26.96 (±2.24) after education (post-test) to 23.05(±2.80) at three months, which corresponds to a reduction of 13.6% of the proportion of correct answers from 93.0% to 79.5% (Table 4). However, the knowledge scores remained significantly (V = 393, P<0.001) higher than the baseline (pre-test) knowledge score where the average score was only 19.98 (±2.72) which correspond to 68.9% correct answers (Table 4). In other words, students retained the rabies knowledge with a 10.6% higher score at three months compared to the base line knowledge on day 0 (pre-test). There was a slight increase in mean knowledge scores in the control groups from a T0 score of 20.14 (±5.06) to 21.34 (±3.44) at three months although this difference was not significant (V = 175.5, P = 0.157) (Table 4). When the answers were assessed individually, significant reductions of the proportions of correct answers were observed at three months (S4 Table) although the average scores remained higher than the baseline scores (S4 Table).

When the intervention group and control groups were compared at three months, there was a significant (P = 0.015) difference in the mean scores, with a higher percentage of correct answers in the intervention compared to the control groups (Table 2). The comparison of correct answers related to knowledge between intervention and control groups at three months is presented in S4 Table.

#### Perception scores

The results are similar to those of the knowledge level, with a significant (V = 2762.5, P<0.001) reduction of students’ perceptions observed, with mean scores reducing from 13.95 (±1.36) at post-test to 12.21 (±2.20) which corresponds to a reduction of correct answers from 99.6% to 87.2% at three months. But the mean scores remain significantly (V = 734, P<0.001) higher than the baseline scores of 10.77 (±1.89 with 72.6% correct answers which is 14.6% higher than baseline scores at day 0 (pre-test) (Table 4).

In the control groups, the mean scores increased from 10.17 (±2.38 with 72.6% correct answers to 11.2 (±2.44 and 80.0% correct answers), with a significant (V = 144.5, P = 041) change of 7.3% in the proportions of correct answers (Table 4). Although there was a significant reduction of correct answers at three months when compared to the proportion of correct answers at post-test, the proportion of correct answers was significantly higher at three months compared to the scores obtained during pre-test (Table 4). When the mean scores of interventions and control groups were compared at three-month periods, significant (W = 1255, P = 0.037) differences were observed between the two groups, with a higher percentage of correct answers in the intervention groups compared to the control group (Table 2). The detailed results of the comparisons of proportion of correct answers at three months period between intervention (retention) and control group (Control T2) are presented in S4 Table.

#### Dog bites safety behaviour

Regarding the dog bite safety behaviour, the mean scores immediately after the education was 12.97(±1.54) which decreased significantly (V = 1617, P = 0.023) to 12.41(±1.64) at three months, which corresponds to a reduction of the proportion of correct answers from 86.5% to 82.7%. However, the mean score remained significantly higher compared to the baseline scores (pre-test) of 9.809±1.85, where the students answered 65.4% of the questions correctly (V = 145.5, P<0.001) (Table 4).

In the control group, the mean scores of the knowledge before the rabies education intervention (Control T0) increased from 9.143 (±1.44) to 10.74 (±1.95) at three months (Control T2), which corresponds to an increase of correct answers from 60.9% to 71.6% (Table 4). On individual question, there was no significant reduction of knowledge related to dog bite safety behaviour at three months (S4 Table). When the answers were compared with baseline knowledge (Control T0), we found a significantly higher proportion of correct answers observed at three months (S4 Table).

When the mean scores were compared between the intervention group (retention) and the control group (control T2) at three months, there was a significant difference observed in the knowledge level between the two groups (Table 2).

## Discussion

The objective of this study was to assess the immediate impacts of rabies awareness education and to find out how long rabies information was retained by the school children in Bhutan. To our knowledge, this is the first study conducted in the country to evaluate the effectiveness of rabies education in terms of immediate assimilation and retention of the information by the students.

Our study demonstrated that rabies knowledge of school children increased dramatically after a short rabies lesson with power point presentation. Although significantly reduced at three months of assessment, the information was largely retained by the students even after three months of intervention (V = 224, P<0.001). Similar to our study, knowledge retention for more than baseline knowledge level 9 weeks after rabies education had been reported in Malawi [11]. This indicated that the rabies lesson provided to the students was effective in terms of improving the knowledge, perceptions and understanding of dog bites safety behaviour, as intended. Using similar kind of education tools, studies conducted in India have reported positive impacts after education program in school children studying in similar grades [10,15]. For instances, overall score improvement of 19% was reported by students studying in neighboring Indian states of Sikkim [15] which is similar with the overall score improvement of 22.4% (from 68.7% to 91.1%) in our study. Improvements of rabies knowledge were also demonstrated in children using different education tools such as teaching lessons as part of the school curriculum in the Philippines [13,14], video and role-playing with life-size toy dogs in USA [16], live dog in Australia [17] and using photographs or styled drawings in Australia [31]. However, whether the improvement in the theoretical understanding would translate into the real practice by the children needs to be further assessed. Moreover, the relevancy and impacts of other education tools need to be evaluated in our context. The study by Chapman et al [17] in pre-school children in Australia found that educational lessons are beneficial when children were taught by interacting with a real dogs. Their study found out that children who receive lessons were more cautious when approaching dogs compared to those that did not attend the lessons. Therefore, although our study did not assess the practical interactions of the school children with the dogs, we assume that improvements in knowledge level will bring the necessary changes in the real practices. Although we observed slight increases in the proportion of correct answers at three months in the control groups, the changes observed were not very large, as in the intervention groups where rabies lessons were provided. Moreover, before the rabies education, knowledge levels were almost same for both the intervention and control groups. This further demonstrate that rabies lessons improve the students’ knowledge on rabies. A slight improvement in the knowledge levels among control groups at three months might be due to sharing of the information by friends who attended the rabies lessons (by intervention group students) (Table 4).

The majority of the students were able to correctly identify dog as the main source and dog bites as main transmission route of rabies. Understanding these information is very important since dog bites are responsible for 99% of rabies deaths in the world [2]. The proportion of correct answers significantly improved after the education and was found to remain nearly intact up to three months. However, not all students knew the source and route of transmission of rabies even after the lesson. For instance, one student (immediately after the lesson -post-test)—and three students at three months (retention test) of survey did not correctly answer these questions, which can be very risky. This may be because we used only power point slides to teach on rabies and they might not have assimilated this point or were not attentive during presentation. Therefore, a combination of education tools, for examples, demonstrating via leaflets, pictures, videos and lectures could be helpful in improving the effectiveness of the rabies lessons. The children’s knowledge of dog bites as the main transmission route of rabies is important so that they take necessary precautionary measures to protect themselves from the bites. Generally, children tend to underestimate the danger arising from dogs because they are more careless and inexperienced compared with the adults during their interaction with dogs [32]. They often do not understand dog’s facial expressions and can confuse a fearful or angry dog with a friendly one thus resulting in dog bites [6]. Understanding that dog bites can be fatal, could help them in their interactions with dogs, such as not approaching the dogs while eating or disturbing them while sleeping, which were reported factors triggering up to 86% of dog bites at home [33]. Although the majority of the students in our study understood prevention tips such as not kicking the dogs when they see dog on the roadside and not disturbing them when eating food or sleeping (S2 Table), some students believed that it was safe to feed the dogs, throw stones at them, play with puppies, and run away if the dog approached or started biting them (S2 Table) which are not appropriate behaviors and can trigger bites. However, there were significant improvements after the lesson and most of the information was retained up to three months which was a significant achievement of this awareness education (S3 Table). Similar improvement in bites prevention behaviors after different lessons in children have been reported in other studies [16,33]. We have also observed that the students’ knowledge in relation to other source of rabies transmission have significantly improved immediately after the education. However, most of the information were not retained when assessed at three months. Although rabies transmission in humans from domestic animals was never reported in Bhutan, 51.5% of animal rabies cases are still reported in cattle via dog bites and thus, children need to take precaution to prevent exposure to rabid domestic animals and livestock [34].

Although rabies is 100% fatal after showing clinical signs, thorough washing of the bite wound with soap and water and receiving timely post-exposure prophylaxis (PEP) can prevent rabies infection [2,35]. However, these simple measures are not followed in rabies endemic countries, resulting in fatal cases [10,36–38]. Lack of awareness on the importance of visiting health centres/clinics was one of the main reasons for not seeking PEP [2,39]. In Bhutan, underestimating the risk as minor if bitten by pet dogs has been identified as one of the main reasons for not visiting the hospital following a dog bite [40]. In our study, the majority of the students understood the importance of washing the bite wound with soap and water (85.1%, n = 80) and visiting hospital after dog bites (97.9%, n = 92) even before our rabies lesson (pre-test). The percent of student understanding this increased to 100% after the rabies safety. However, this knowledge was not retained by all the students when assessed at three months since only 93.6% (n = 88) had retained the information up to three months (S3 Table). This underscores the need for frequent awareness program among the students, possibly as often as 3–4 times per year in rabies endemic areas of the country. The Philippines [13] and Sri Lanka [14] had integrated rabies education in the school curriculum, and this could be the way forward for improving rabies education among school children in Bhutan as well.

Our study also explored factors associated with knowledge levels of the students. Older students (>16 years), students whose mothers were employed, and students studying in SJMSS (border town) were found to have more knowledge about rabies. The association of higher knowledge with the older age groups has been reported in many studies [11,33]. Higher knowledge by the students of SMSS may be due to reports of frequents outbreaks of rabies in the town areas and also due to awareness and mass dog vaccination campaign [24]. The higher level of knowledge of students whose mothers are employed may be due to the better accessibilities to the rabies education materials by educated employed mothers, who may be in a better position to share this information with their children. A previous study carried out in Bhutan on knowledge, attitude and practice study among school children in rabies endemic areas also observed similar findings [41].

The main limitation of the current study is that we were not able to carry out follow up surveys at six months and one-year periods as initially planned, due to COVID-19 pandemic restrictions. A follow up surveys would have provided information about the retention capacity of the students so that intervention /awareness education program can be planned accordingly. Nevertheless, our study provides some baseline information about the students’ assimilation and retention capacity of the rabies lessons. Another limitation of our study was the teaching materials/methods used. We employed only a power point presentation and could not compare with other teaching aids tools due to COVID-19 restrictions. We believe that a combination of several teaching aids such as posters, role play, game and drama, video, lecture, group works etc., would improve the understanding and retention time by the students compared to a single tool. Therefore, a separate study incorporating these education tools would be necessary for obtaining better data so that an evidence-based rabies lessons can be developed for children education program.

## Conclusion

Our study indicates that awareness education program helps in improving the knowledge levels and safety behaviours of the school children. But the knowledge level significantly dropped when assessed at three months after the lesson was given, indicating the need for frequent awareness programs, at least quarterly or half yearly. There is also a need to focus on critical points such as dog and dog bites are the main source of rabies, the importance of washing a dog bite wound with soap and water, and importance of visiting the hospital for medical advice following animal bites during awareness program. Dog safety behaviours should also be taught to the students since children are at high risk of dog bite incidents. The results also indicate the needs for targeting the awareness campaign to the younger children and children belonging to low-income families.

## Supporting information

S1 Table Scoring system. (DOCX)

S2 Table Baseline knowledge, perception and safety behaviour of students (frequency of positive response). (DOCX)

S3 Table Change in knowledge level of students in the intervention groups after rabies education. (DOCX)

S4 Table Comparison of knowledge between intervention group (n = 94) and control group (n = 35) before education and 12 weeks after education program. (DOCX)

S1 File Questionnaires. (DOCX)
