# Supplementary material for: Assessment of the rabies education among middle secondary school students of southeastern Bhutan
Source: PLoS One. 2022 Dec 12;17(12):e0276862. doi: 10.1371/journal.pone.0276862 (PMC9744285; doi:10.1371/journal.pone.0276862)
Supplement: S1 Table — (DOCX) [file pone.0276862.s001.docx]

**S1 Table. Scoring system**

| **Questions** | **Answers** | **Scores*** |
| --- | --- | --- |
| Do you know that rabies is present in Bhutan? | Yes/No | (1/0) |
| Which animals do you think will get rabies? | Dog (Yes/No) | (2/0) |
|  | Pig (Yes/No) | (1/0) |
|  | Cat (Yes/No) | (1/0) |
|  | Cow (Yes/No) | (1/0) |
|  | Snake (Yes/No) | (0/1) |
|  | Tiger (Yes/No) | (1/0) |
|  | Bats (Yes/No) | (1/0) |
|  | Birds (Yes/No) | (0/1) |
| Name one animal that is the most important source of rabies? | Dog /others | (2/0) |
| Can human get rabies? | Yes/No/I don’t know | (2/0/0) |
| What do think are the common routes of rabies transmission? | Dog bite ((Yes/No) | (2/0) |
|  | Scratches by dog and cats (Yes/No) | (1/0) |
|  | Licks (Yes/No) | (1/0) |
|  | From touching the animals (Yes/No) | (1/0) |
|  | Contact with urine and faeces of animals (Yes/No) | (0/1) |
| What are the common symptoms of rabies in dogs? | Become aggressive and bite anything (Yes/No) | (2/0) |
|  | Salivation from mouth (Yes/No) | (1/0) |
|  | Abnormal barking (Yes/No) | (1/0) |
|  | Leg paralysis and unable to walk/move (Yes/No) | (1/0) |
|  | Diarrhoea (Yes/No) | (0/1) |
|  | Vomiting (Yes/No) | (0/1) |
| What is the schedule of rabies vaccine injection in human given after dog bite? | Day 0, day 3, day 7, day 28 (Yes/No) | (2/0) |
|  | Day 0, day 3, 28 days (Yes/No) | (0/0) |
|  | Day 0, day 3 (Yes/No) | (0/0) |
|  | Day 0 (Yes/No) | (0/0) |
| **Total knowledge score** | | **29** |
| What do you think are the methods to prevent dogs from getting rabies? | By giving rabies vaccine injection (Yes/No) | (1/0) |
|  | By operating the dogs (Yes/No) | (0/0) |
|  | By washing the dog with shampoo (Yes/No) | (0/0) |
|  | By giving them food everyday (Yes/No) | (0/0) |
| What will happen if people get rabies? | No treatment and die (Yes/No) | (2/0) |
|  | Recover after treatment (Yes/No) | (0/0) |
| What should you do if you are bitten by dog or cats? | I will wash the wound with water and soap for 10-15 minutes (Yes/No) | (2/0) |
|  | I will report to parents/teachers (Yes/No) | (1/0) |
|  | I will go to hospital (Yes/No) | (2/0) |
|  | I will cover the bite wound with cloth (Yes/No) | (0/1) |
|  | I will hide the wound and not inform to anyone and also not visit the hospital (Yes/No) | (0/1) |
| What should you do if you see a dog looking sick or showing abnormal behaviors in the town or your school campus? | I will catch and take the dog to animal hospital for treatment | (0/1) |
|  | I will report to teachers and parents | (1/0) |
|  | I will report to animal/livestock staff | (1/0) |
|  | I will inform /alert the nearby people | (1/0) |
|  | I will not do anything | (0/0) |
| **Total perception score** | | **14** |
| If a strange dog comes near you, stand still like a tree without moving and do not run away | True /False | (1/0) |
| Kick the dogs when you see them on road or school or in the town(false) | True /False | (0/1) |
| Throw stones and objects at the dogs to chase them away (false) | True /False | (0/1) |
| You can go near and disturb the dogs when they are eating food | True /False | (0/1) |
| It is safe to play with the puppies when the mother is feeding them | True /False | (0/1) |
| It is very safe to play with puppies or young dog than adult dog | True /False | (0/1) |
| Wake up the dog when you find them sleeping | True /False | (0/1) |
| Go near and separate the dogs when you see them fighting | True /False | (0/1) |
| Cover your face/head with *Gho or Tego* and scroll down to the ground if a dog started biting you | True /False | (1/0) |
| Runaway fast if a dog started biting you | True /False | (0/1) |
| Call the pack of dogs and give your leftover food (lunch) to the dogs | True /False | (0/1) |
| Wash the hands after toughing or playing with the dogs | True /False | (1/0) |
| When dog is angry, they show their teeth, growl and pull their tail straight up in the air | True /False | (1/0) |
| Dog should be approached slowly and confidently, let them sniff your hand and pet them on back before touching | True /False | (1/0) |
| Dog bite in the face is more dangerous than bite on the leg | True /False | (1/0) |
| **Total dog bite safety scores** | | **15** |
| Note= *2=completely correct answers, 1=correct answers, 0=wrong answers | | |
