# Supplementary material for: Assessment of the rabies education among middle secondary school students of southeastern Bhutan
Source: PLoS One. 2022 Dec 12;17(12):e0276862. doi: 10.1371/journal.pone.0276862 (PMC9744285; doi:10.1371/journal.pone.0276862)
Supplement: S2 Table — (DOCX) [file pone.0276862.s002.docx]

**S2 Table. Baseline knowledge, perception and safety behaviors of students (frequency of positive response)**

| **Positive responses** | **Total (n=129)** | **Groups** | |  |
| --- | --- | --- | --- | --- |
|  |  | **Intervention (n=94)** | **Control (n=35)** | **P value** |
| **Knowledge** |  |  |  |  |
| Know rabies is present in Bhutan | 102 (79.1) | 73(77.7) | 29(82.9) | 0.688 |
| Susceptible host of rabies |  |  |  |  |
| Dog | 126(97.7) | 93(98.9) | 33(94.3) | 0.179 |
| Pig | 33(25.6) | 16(17.0) | 17(48.6) | **0.001** |
| Cat | 81(62.8) | 27(77.1) | 54(57.4) | 0.040 |
| Cow | 33 (25.6) | 13(57.1) | 20(13.8) | **<0.001** |
| Snake | 12(8.5) | 8(8.5) | 4(11.4) | 0.418 |
| Tiger | 29(22.5) | 11(11.7) | 18(51.4) | **<0.001** |
| Bats | 46(35.7) | 24 (62.9) | 22(25.5) | **<0.001** |
| Birds | 28(21.7) | 15(16.0) | 13(37.1) | **0.009** |
| Know dogs as most important source of rabies | 124(96.1) | 91 (96.8) | 33(94.3) | 0.372 |
| Know human can get rabies | 98(76.0) | 71(75.5) | 27(77.1) | 0.716 |
| Rabies transmission route |  |  |  |  |
| Dog bite | 115(89.1) | 88(93.6) | 27(77.1) | **0.019** |
| Scratches by dog and cats | 81(62.8) | 59(62.8) | 22 62.9 | 0.992 |
| Licks | 55(42.6) | 37(39.4) | 18(51.4) | 0.218 |
| From touching the animals | 37(28.7) | 27(28.7) | 10(28.6) | 0.986 |
| Contact with urine and faeces of animals | 15(11.6) | 12(12.8) | 3(8.6) | 0.376 |
| Clinical signs of rabies in animals |  |  |  |  |
| Become aggressive and bite anything | 93(72.1) | 72(76.6) | 21(60.00) | 0.099 |
| Salivation from mouth | 99(76.7) | 73(77.7) | 26(74.3) | 0.866 |
| Abnormal barking | 73(56.6) | 53(56.4) | 20(57.1) | 0.938 |
| Leg paralysis and unable to walk/move | 41(31.8) | 37(39.4) | 4(11.4) | **0.005** |
| Diarrhea | 11(8.5) | 10(10.6) | 1(2.9) | 0.145 |
| Vomiting | 35(27.1) | 30(31.9) | 5(14.3) | 0.075 |
| Schedule of vaccination | 81(62.8) | 68(72.3) | 13(37.1) | **0.002** |
| **Perceptions and beliefs** |  |  |  |  |
| Believe that rabies in dog can be prevented by administering rabies vaccine | 115(89.1) | 81(86.2) | 34(97.1) | 0.253 |
| Believe that rabies have no treatment after showing clinical signs | 11(8.5) | 9(9.6) | 2(5.7) | **0.043** |
| Believe that following things should be done if bitten by dogs |  |  |  |  |
| I will wash the wound with water and soap for 10-15 minutes | 106(82.2) | 80(85.1) | 26(74.3) | 0.242 |
| I will report to parents/teachers | 98(76.0) | 78(83.0) | 20(57.1) | **0.005** |
| I will go to hospital | 124(96.1) | 92(97.9) | 32(91.4) | 0.123 |
| I will cover the bite wound with cloth | 19(14.7) | 18(94.7) | 1(5.3) | **0.041** |
| I will hide the wound and not inform to anyone and also not visit the hospital | 2(1.6) | 2(2.1) | 0(0.0) | 0.529 |
| I will not do anything | 1(0.8) | 1(1.1) | 0(0.0) | 0.729 |
| Believe that following things should be done if they see rabid dogs in street |  |  |  |  |
| I will catch and take the dog to animal hospital for treatment 0 | 61(47.3) | 52(55.3) | 9(25.7) | **0.005** |
| I will report to teachers and parents | 69(53.5) | 57(60.6) | 12(34.3) | **0.014** |
| I will report to animal/livestock staff | 99(76.7) | 77(81.9) | 22(62.9) | **0.041** |
| I will inform /alert the nearby people | 85(65.9) | 66(70.2) | 19(54.3) | 0.137 |
| I will not do anything | 2(1.6) | 1(1.1) | 1 (2.9) | 0.471 |
| **Safety behaviors** |  |  |  |  |
| If a strange dog comes near you, stand still like a tree without moving and do not run away | 56(43.4) | 49(52.1) | 7(20.0) | **0.002** |
| Kick the dogs when you see them on road or school or in the town(false) | 10(7.8) | 5(5.3) | 5(14.3) | 0.097 |
| Throw stones and objects at the dogs to chase them away | 59(45.7) | 43(45.7) | 16(45.7) | 0.998 |
| You can go near and disturb the dogs when they are eating food | 4(3.1) | 4(4.3) | 0(0.0) | 0.574 |
| It is safe to play with the puppies when the mother is feeding them | 12(9.3) | 10(10.6) | 2(5.7) | 0.316 |
| It is very safe to play with puppies or young dog than adult dog | 94(72.9) | 69(73.4) | 25(71.4) | 0.999 |
| Wake up the dog when you find them sleeping | 14(10.9) | 13(13.8) | 1(2.9) | 0.063 |
| Go near and separate the dogs when you see them fighting | 55(42.6) | 34(36.2) | 21(60.0) | **0.026** |
| Cover your face/head with shirt or cloths and scroll down to the ground if a dog started biting you | 61(47.3) | 46(48.9) | 15(42.9) | 0.677 |
| Runaway fast if a dog started biting you | 105(81.4) | 73(77.7) | 32(91.4) | **0.057** |
| Call the pack of dogs and give your leftover food (lunch) to the dogs | 92(71.3) | 66(70.2) | 26(74.3) | 0.814 |
| Wash the hands after toughing or playing with the dogs | 120(93.0) | 89(94.7) | 31(88.6) | 0.201 |
| When dog is angry, they show their teeth, growl and pull their tail straight up in the air | 106(82.2) | 79(84.0) | 27(77.1) | 0.515 |
| Dog should be approached slowly and confidently, let them sniff your hand and pet them on back before touching | 76(58.9) | 54(57.4) | 22(62.9) | 0.723 |
| Dog bite in the face is more dangerous than bite on the leg | 107(82.9) | 76(80.9) | 31(88.6) | 0.223 |
