# Supplementary material for: Assessment of the rabies education among middle secondary school students of southeastern Bhutan
Source: PLoS One. 2022 Dec 12;17(12):e0276862. doi: 10.1371/journal.pone.0276862 (PMC9744285; doi:10.1371/journal.pone.0276862)
Supplement: S3 Table — (DOCX) [file pone.0276862.s003.docx]

**S3 Table. Change in knowledge level of students in the intervention groups after rabies education lesson**

| **Positive response** | **Intervention group** | | |  | **Significance’s level** | | |
| --- | --- | --- | --- | --- | --- | --- | --- |
|  | **Pretest** | **Post test** | **retention** |  | **Pre Vs post** | **Post Vs Ret** | **Ret Vs Pre** |
| **Section A: Knowledge** |  |  |  |  |  |  |  |
| Know rabies is present in Bhutan | 73(77.7) | 92(97.9) | 85(90.4) |  | **<0.001** | **0.029** | **0.029** |
| Susceptible host of rabies |  |  |  |  |  |  |  |
| Dog | 93 (98.9) | 94 (100) | 94 (100) |  | NA | NA | NA |
| Pig | 16 (17) | 86 (91.5) | 54 (57.4) |  | **<0.001** | **<0.001** | **<0.001** |
| Cat | 54 (57.4) | 92 (97.9) | 74(78.7) |  | **<0.001** | **<0.001** | **0.003** |
| Cow | 13 (13.8) | 90(95.7) | 50(53.2) |  | **<0.001** | **<0.001** | **<0.001** |
| Snake | 8(8.5) | 3(3.2) | 5(5.3) |  | 0.106 | 0.36 | 0.565 |
| Tiger | 11(11.7) | 82(87.2) | 39(41.5) |  | **<0.001** | **<0.001** | **<0.001** |
| Bats | 24(25.5) | 78(25.5) | 46(25.5) |  | **<0.001** | **<0.001** | **0.002** |
| Birds (no) | 15(16.0) | 1(1.1) | 17(18.1) |  | **0.000** | **<0.001** | 0.846 |
| Know dogs as most important source of rabies | 91(96.8) | 94(100.0) | 92 (97.9) |  | 0.246 | 0.497 | 0.497 |
| Know human can get rabies | 71(75.5) | 88(93.6) | 80(85.1) |  | **<0.001** | 0.154 | **0.000** |
| Rabies transmission routes |  |  |  |  |  |  |  |
| Dog bite | 88(93.6) | 93(98.9) | 91(96.8) |  | **0.059** | 0.31 | 0.249 |
| Scratches by dog and cats | 59(62.8) | 87(92.6) | 68(72.3) |  | **<0.001** | **0.001** | 0.213 |
| Licks | 37(39.4) | 72(76.6) | 59(62.8) |  | **<0.001** | **0.057** | **0.002** |
| From touching the animals | 27(28.7) | 13(13.8) | 15(16.0) |  | **0.013** | 0.838 | **0.054** |
| Contact with urine and feces of animals | 12(12.8) | 9(9.6) | 16(17.0) |  | 0.643 | 0.197 | 0.539 |
| Clinical signs of rabies in animals |  |  |  |  |  |  |  |
| Become aggressive and bite anything | 72(76.6) | 83(88.3) | 71(75.5) |  | **0.035** | **0.037** | 1 |
| Salivation from mouth | 73(77.7) | 90(95.7) | 92(97.9) |  | **0.000** | 0.341 | **<0.001** |
| Abnormal barking | 53(56.4) | 77(81.9) | 68(81.9) |  | **0.000** | 0.165 | **0.033** |
| Leg paralysis and unable to walk/move | 37(39.4) | 74(78.7) | 53(56.4 ) |  | **<0.001** | **0.002** | **0.029** |
| Diarrhea | 10(10.6) | 2(2.1) | 5(5.3) |  | **0.016** | 0.222 | 0.282 |
| Vomiting | 30(31.9) | 6(6.4) | 41(43.6) |  | **<0.001** | **<0.001** | 0.132 |
| Schedule of vaccination | 68(72.3) | 90(95.7) | 79(84.0) |  | **<0.001** | **0.017** | **0.062** |
| **Section B: Attitude** |  |  |  |  |  |  |  |
| Believe that rabies in dog can be prevented by administering rabies vaccine | 81(86.2) | 90(95.7) | 76(80.9) |  | 0.171 | **0.016** | 0.678 |
| Believe that rabies have no treatment after showing clinical signs | 9(9.6) | 81(86.2) | 57(60.6) |  | **<0.001** | **0.000** | **<0.001** |
| Believe that following things should be done if bitten by dogs |  |  |  |  |  |  |  |
| I will wash the wound with water and soap for 10-15 minutes | 80(85.1) | 94(100) | 88(93.6) |  | **0.000** | **0.038** | 0.098 |
| I will report to parents/teachers | 78(83.0) | 89(94.7) | 78(83.0) |  | **0.021** | **0.021** | 1 |
| I will go to hospital | 92(97.9) | 91(96.8) | 88(93.6) |  | 0.5 | 0.249 | 0.139 |
| I will cover the bite wound with cloth | 18(19.1) | 11(11.7) | 65(69.1) |  | 0.226 | **0.002** | 0.092 |
| I will hide the wound and not inform to anyone and also not visit the hospital | 2(2.1) | 0(0.0) | 94(100.0) |  | 0.249 | NA | 0.249 |
| I will not do anything | 1(1.1) | 0(0.0) | 94(100.0) |  | 0.5 | NA | 0.5 |
| Believe that following things should be done if they see rabid dogs in street |  |  |  |  |  |  |  |
| I will catch and take the dog to animal hospital for treatment 0 | 52(55.3) | 2(2.1) | 19(20.2) |  | **<0.001** | **0.000** | **<0.001** |
| I will report to teachers and parents | 57(60.6) | 75(79.8) | 67(69.1) |  | **<0.001** | 0.132 | 0.285 |
| I will report to animal/livestock staff | 77(81.9) | 91(96.8) | 87(92.6) |  | **0.002** | 0.33 | **0.049** |
| I will inform /alert the nearby people | 66(70.2) | 75(79.8) | 66(70.2) |  | 0.178 | 0.178 | 1 |
| I will not do anything | 1(1.1) | 1(1.1) | 1(1.1) |  | 1(0.8) | 0.751 | 0.751 |
| **Section C: Safety behaviors** |  |  |  |  |  |  |  |
| If a strange dog comes near you, stand still like a tree without moving and do not run away | 49(52.1) | 92(97.9) | 87(92.6) |  | **<0.001** | 0.085 | **<0.001** |
| Kick the dogs when you see them on road or school or in the town(false) | 5(5.3) | 0(0.0) | 2 (2.1) |  | **0.059** | 0.497 | 0.222 |
| Throw stones and objects at the dogs to chase them away (false) | 43(45.7) | 10(10.6) | 14(14.9) |  | **<0.001** | 0.512 | **<0.001** |
| You can go near and disturb the dogs when they are eating food | 4(4.3) | 2(2.1) | 1(1.10 |  | 0.341 | 0.877 | 0.184 |
| It is safe to play with the puppies when the mother is feeding them | 10(10.6 | 3(3.2) | 3(3.2) |  | **0.041** | 0.659 | **0.041** |
| It is very safe to play with puppies or young dog than adult dog | 69(73.4) | 60(63.8) | 65(69.1) |  | 0.209 | 0.537 | 0.629 |
| Wake up the dog when you find them sleeping | 13(13.8) | 4(4.3) | 1(1.1) |  | **0.020** | 0.184 | **0.001** |
| Go near and separate the dogs when you see them fighting | 34(36.2) | 8(8.5) | 9(9.6) |  | **<0.001** | 1 | **<0.001** |
| Cover your face/head with shirt or cloth and scroll down to the ground if a dog started biting you | 46(48.9) | 85(90.4) | 87(92.6) |  | **<0.001** | 0.794 | **<0.001** |
| Runaway fast if a dog started biting you | 73(77.7) | 7(18.1) | 33 (35.1) |  | **<0.001** | **0.013** | **<0.001** |
| Call the pack of dogs and give your leftover food (lunch) to the dogs | 66(70.2) | 32(34.0) | 39(41.5) |  | **<0.001** | 0.367 | **0.000** |
| Wash the hands after toughing or playing with the dogs | 89(94.7) | 94(100) | 92(97.9) |  | **0.030** | 0.249 | 0.222 |
| When dog is angry, they show their teeth, growl and pull their tail straight up in the air | 79(84.0) | 82(87.2) | 81(86.2) |  | 0.677 | 1 | 0.838 |
| Dog should be approached slowly and confidently, let them sniff your hand and pet them on back before touching | 54(57.4) | 68(72.3) | 58(61.7) |  | **0.047** | 0.163 | 0.656 |
| Dog bite in the face is more dangerous than bite on the leg | 76(80.9) | 88(93.6) | 83(88.3) |  | **0.007** | 0.309 | 0.226 |
