# Supplementary material for: Assessment of the rabies education among middle secondary school students of southeastern Bhutan
Source: PLoS One. 2022 Dec 12;17(12):e0276862. doi: 10.1371/journal.pone.0276862 (PMC9744285; doi:10.1371/journal.pone.0276862)
Supplement: S4 Table — (DOCX) [file pone.0276862.s004.docx]

**S4 Table: Comparison of knowledge between intervention group (n=94) and control group (n=35) before education and 12 weeks after education program**

| **Positive responses** | **Pre-education assessment** | | | **Assessment after 12 weeks** | | |
| --- | --- | --- | --- | --- | --- | --- |
|  | **Intervention (n=94)** | **Control (n=35)** | **P value** | **Intervention (n=94)** | **Control (n=35)** | **P value** |
| **Knowledge** |  |  |  |  |  |  |
| Know rabies is present in Bhutan | 73(77.7) | 29(82.9) | 0.688 | 85(90.4) | 30(85.7) | 0.317 |
| Susceptible host of rabies |  |  |  |  |  |  |
| Dog | 93(98.9) | 33(94.3) | 0.179 | 94 (100) | 35(100.0) | NA |
| Pig | 16(48.6) | 17(17.0) | **0.001** | 54 (57.4) | 12(34.3) | **0.032** |
| Cat | 27(77.1) | 54(57.4) | **0.040** | 74(78.7) | 26(74.3) | 0.764 |
| Cow | 13(57.1) | 20(13.8) | **<0.001** | 50(53.2) | 15(42.9) | 0.398 |
| Snake | 8(8.5) | 4(11.4) | 0.418 | 5(5.3) | 2(5.7) | 0.612 |
| Tiger | 11(11.7) | 18(51.4) | **<0.001** | 39(41.5) | 10(28.6) | 0.254 |
| Bats | 24 62.9 | 22(25.5) | **<0.001** | 46(25.5) | 17(48.6) | 1 |
| Birds (no) | 15(16) | 13(37.1) | **0.009** | 17(18.1) | 10(28.6) | **0.29** |
| Know dogs as most important source of rabies | 91 96.8 | 33 94.3 | 0.372 | 92 (97.9) | 33(94.3) | 0.297 |
| Know human can get rabies | 71(75.5) | 27(77.1) | 0.716 | 80(85.1) | 28(80.0) | 0.613 |
| Transmission |  |  |  |  |  |  |
| Dog bite | 88(93.6) | 27(77.1) | **0.019** | 91(96.8) | 33(94.3) | 0.413 |
| Scratches by dog and cats | 59(62.8) | 22 62.9 | 0.992 | 68(72.3) | 21(60.0) | 0.257 |
| Licks | 37(39.4 ) | 18(51.4) | 0.218 | 59(62.8) | 21(60.0) | 0.933 |
| From touching the animals | 27(28.7) | 10(28.6) | 0.986 | 15(16.0) | 5(14.3) | 1 |
| Contact with urine and feces of animals | 12(12.8) | 3(8.6) | 0.376 | 16(17.0) | 4(11.4) | 0.612 |
| Clinical signs |  |  |  |  |  |  |
| Become aggressive and bite anything | 72(76.6) | 21(60.00) | 0.099 | 71(75.5) | 27(77.1) | 1 |
| Salivation from mouth | 73(77.7) | 26(74.3) | 0.866 | 92(97.9) | 28(80.0) | **0.002** |
| Abnormal barking | 53(56.4) | 20(57.1) | 0.938 | 68(81.9) | 24(68.6) | 0.84 |
| Leg paralysis and unable to walk/move | 37(39.4) | 4(11.4) | **0.005** | 53(56.4) | 12(34.3) | **0.042** |
| Diarrhea | 10(10.6) | 1(2.9) | 0.145 | 5(5.3) | 8(22.9) | **0.007** |
| Vomiting | 30(31.9) | 5(14.3) | 0.0751 | 41(43.6) | 14(40.0) | 0.866 |
| Schedule of vaccination | 68(72.3) | 139(37.1) | **0.002** | 79(84.0) | 26(74.3) | 0.121 |
| **Perception** |  |  |  |  |  |  |
| Believe that rabies in dog can be prevented by giving vaccination | 81(86.2) | 34(97.1) | 0.253 | 76(80.9) | 33(94.3) | 0.454 |
| Believe that rabies had no treatment after showing clinical signs | 9(9.6) | 2(5.7) | **0.043** | 57(60.6) | 19(54.3) | 0.781 |
| Believe that following things should be done if bitten by dogs |  |  |  |  |  |  |
| I will wash the wound with water and soap for 10-15 minutes | 80(85.1) | 26(74.3) | 0.242 | 88(93.6) | 31(88.6) | 0.27 |
| I will report to parents/teachers | 78(83.0) | 20(57.1) | **0.005** | 78(83.0) | 22(62.9) | **0.028** |
| I will go to hospital | 92(97.9) | 32(91.4) | 0.123 | 88(93.6) | 34(97.1) | 0.388 |
| I will cover the bite wound with cloth | 18(94.7) | 1(5.3) | **0.041** | 65(69.1) | 10(28.6) | 0.972 |
| I will hide the wound and not inform to anyone and also not visit the hospital | 2(2.1) | 0 | 0.529 | 94(100.0) | 35(100) | NA |
| I will not do anything | 1(1.1 | 0 | 0.729 | 94(100) | 35(100) | NA |
| Believe that following things should be done if they see rabid dogs in street |  |  |  |  |  |  |
| I will catch and take the dog to animal hospital for treatment | 52(55.3) | 9(25.7) | **0.005** | 19(20.2) | 19(54.3) | **0.000** |
| I will report to teachers and parents | 57(60.6) | 12(34.3) | **0.014** | 67(69.1) | 16(45.7) | **0.025** |
| I will report to animal/livestock staff | 77(81.9) | 22(62.9) | **0.041** | 87(92.6) | 26(74.3) | **0.013** |
| I will inform /alert the nearby people | 66(70.2) | 19(54.3) | 0.137 | 66(70.2) | 18(51.4) | 0.075 |
| I will not do anything | 1(1.1) | 1 (2.9) | 0.471 | 1(1.1) | 0(0.00) | 0.729 |
| **Safety behaviors** |  |  |  |  |  |  |
| If a strange dog comes near you, stand still like a tree without moving and do not run away | 49(52.1) | 7(20.0) | **0.002** | 87(92.6) | 23(65.7) | **0.000** |
| Kick the dogs when you see them on road or school or in the town(false) | 5(5.3) | 5(14.3) | 0.097 | 2 (2.1) | 3(8.6) | 0.123 |
| Throw stones and objects at the dogs to chase them away (false) | 43(45.7) | 16(45.7) | 0.998 | 14(14.9) | 15(42.9) | **0.002** |
| You can go near and disturb the dogs when they are eating food | 4(4.3) | 0(0.0) | 0.574 | 1(1.10 | 0(0.00) | 0.729 |
| It is safe to play with the puppies when the mother is feeding them | 10(10.6) | 2(5.7) | 0.316 | 3(3.2) | 1(2.90 | 0.703 |
| It is very safe to play with puppies or young dog than adult dog | 69(73.4) | 25(71.4) | 0.999 | 65(69.1) | 29(82.9) | 0.182 |
| Wake up the dog when you find them sleeping | 13(13.8) | 1(2.9) | 0.063 | 1(1.1) | 1(2.9) | 0.471 |
| Go near and separate the dogs when you see them fighting | 34(36.2) | 21(60.0) | **0.026** | 9(9.6) | 14(40.0) | **0.000** |
| Cover your face/head with *Gho or Tego* and scroll down to the ground if a dog started biting you | 46(48.9) | 15(42.9) | 0.677 | 87(92.6) | 25(71.4) | 0.004 |
| Runaway fast if a dog started biting you | 73(77.7) | 32(91.4) | **0.057** | 33 (35.1) | 26(74.3) | **0.000** |
| Call the pack of dogs and give your leftover food (lunch) to the dogs | 66(70.2) | 26(74.3) | 0.814 | 39(41.5) | 19(54.3) | 0.271 |
| Wash the hands after toughing or playing with the dogs | 89(94.7) | 31(88.6) | 0.201 | 92(97.9) | 35(100) | 0.529 |
| When dog is angry, they show their teeth, growl and pull their tail straight up in the air | 79(84.0) | 27(77.1) | 0.515 | 81(86.2) | 30(85.7) | 0.574 |
| Dog should be approached slowly and confidently, let them sniff your hand and pet them on back before touching | 54(57.4) | 22(62.9) | 0.723 | 58(61.7) | 28(80.0) | 0.080 |
| Dog bite in the face is more dangerous than bite on the leg | 76(80.9) | 31(88.6) | 0.223 | 83(88.3) | 28(80.0) | 0.356 |
