# Supplementary material for: Assessment of the rabies education among middle secondary school students of southeastern Bhutan
Source: PLoS One. 2022 Dec 12;17(12):e0276862. doi: 10.1371/journal.pone.0276862 (PMC9744285; doi:10.1371/journal.pone.0276862)
Supplement: S1 File — (DOCX) [file pone.0276862.s005.docx]

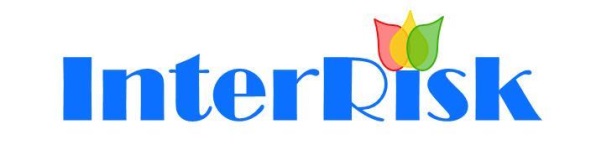


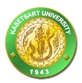

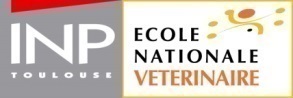

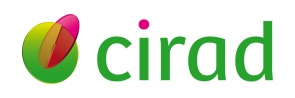


**Assessing the impact of rabies education in school children in south-eastern Bhutan**

| **Informed consent**  The consent form is attached at the back of the questionnaire |
| --- |

Survey No: ­­­­­­­­­­­­­­­­­ …………………………. Date of survey……………….

Name of School: ………………………………. Name of student …………………

Class: ……………. Section: ………………….

**Part I. Respondent information**

| 1.1 | What is your age? | _______years |
| --- | --- | --- |
| 1.2 | Are you a male or female? | ☐Female  ☐Male |
| 1.3 | What is the occupation (job) of your parents? *Select the most important one.*   \| Father’s occupation \| Mother’s occupation \| \| --- \| --- \| \| ☐ Farmer  ☐ Government service  ☐ Military (RBP/RBA/RBG)  ☐ Working in private/corporate office  ☐ Businessman (shopkeeper)  ☐ Others (Specify)…………. \| ☐ Farmer  ☐ Government service  ☐ Military (RBP/RBA/RBG)  ☐ Working in private/corporate office  ☐ Businessman (shopkeeper)  ☐ Others (Specify)……………. \| | |
| 1.4 | How many family members do you have at home? | ……………. |
| 1.5 | Do you have dog at home? | ☐ Yes  ☐ No |
| 1.6 | If Yes, how many dogs do you have? | …………... (Nos) |

**Part II Knowledge and perception on rabies**

| 2.1 | Have you heard about ‘rabies’? | ☐ Yes  ☐ No **If No, go to part III** |
| --- | --- | --- |
| 2.2 | If yes, from where did you hear about rabies?  *(Tick one or more as you think are correct)* | ☐ Parents and family members  ☐ Teachers  ☐ Friends  ☐ Animal health staff  ☐ Health staff  ☐ TV/radio etc  ☐ Newspaper  ☐ Internet |
| 2.3 | Have you attended rabies awareness program in your school in the past 2 years? | ☐ Yes  ☐ No |
| 2.4 | Do you know that rabies is present in Bhutan? | ☐ Yes  ☐ No |
| 2.5 | Which animals do you think will get rabies?  *(Tick as many as you think are correct)* | ☐ Dog  ☐ Pig  ☐ Cat  ☐ Cow  ☐ Snake  ☐ Tiger  ☐ Bats  ☐ Birds  ☐ Others (specify)_________________ |
| 2.6 | Of the above animals, name one animal that is the most important source of rabies? | ____________ |
| 2.7 | Can human get rabies? | ☐ Yes  ☐ No  ☐ I don’t know |
| 2.8 | What do think are the common routes of rabies transmission?  *(Tick as many as you think are correct)* | ☐ Dog bite  ☐ Scratches by dog and cats  ☐ Licks on open wounds by rabid animal  ☐ From touching the animals  ☐ Contact with urine and faeces of animals  ☐ Others (Specify……………..) |
| 2.9 | What are the common symptoms of rabies in dogs?  *(Tick as many as you think are correct)* | ☐ Become aggressive and bite anything  ☐ Salivation from mouth  ☐ Abnormal barking  ☐ Leg paralysis and unable to walk/move  ☐ Diarrhoea  ☐ Vomiting  ☐ Others (specify_____________________) |
| 2.10 | What do you think are the methods to prevent dogs from getting rabies? | ☐ By giving rabies vaccine injection  ☐ By operating the dogs  ☐ By washing the dog with shampoo  ☐ By giving them food everyday  ☐ Other (specify _____________________) |
| 2.11 | What will happen if people get rabies? | ☐ No treatment and die  ☐ Recover after treatment  ☐ I don’t know |
| 2.12 | What should you do if you are bitten by dog or cats?  *(Tick one or more as you think are correct)* | ☐ I will wash the wound with water and soap for 10-15 minutes  ☐ I will report to parents/teachers  ☐ I will go to hospital  ☐ I will cover the bite wound with cloth  ☐ I will hide the wound and not inform to anyone and also not visit the hospital  ☐ I will not do anything  ☐ Others__________________________ |
| 2.13 | Have you been bitten by dog before? | ☐ Yes  ☐ No |
| 2.14 | If yes, have you visited the hospital after the dog bite? | ☐ Yes  ☐ No |
| 2.15 | What is the schedule of rabies vaccine injection in human given after dog bite? | ☐ Day 0, day 3, day 7, day 28  ☐ Day 0, day 3, 28 days  ☐ Day 0, day 3  ☐ Day 0 |
| 2.16 | What should you do if you see a dog looking sick or showing abnormal behaviour in the town or your school campus?  *(Tick one or more as you think are correct)* | ☐ I will catch and take the dog to animal hospital for treatment  ☐ I will report to teachers and parents  ☐ I will report to animal/livestock staff  ☐ I will inform /alert the nearby people  ☐ I will not do anything |

**Part III. Risk behaviours for the dog bite and rabies**

| **3.1** | Do you play with dogs? | ☐ Yes  ☐ No **If No, go to Question no. 3.3** |
| --- | --- | --- |
| **3.2** | If yes, what type of dogs you mostly played with? | ☐ Pet dogs  ☐ Stray dog  ☐ All dog I see |
| **3.3** | Choose either **“True” or “False”** for the following statements based on how you should or should not behave to prevent yourself from being bitten by dogs   \| **Statements** \| **True** \| **False** \| \| --- \| --- \| --- \| \| 1. If a strange dog comes near you, stand still like a tree without moving and do not run away \|  \|  \| \| 1. Kick the dogs when you see them on road or school or in the town \|  \|  \| \| 1. Throw stones and objects at the dogs to chase them away \|  \|  \| \| 1. You can go near and disturb the dogs when they are eating food \|  \|  \| \| 1. It is safe to play with the puppies when the mother is feeding them \|  \|  \| \| 1. It is very safe to play with puppies or young dog than adult dog \|  \|  \| \| 1. Wake up the dog when you find them sleeping \|  \|  \| \| 1. Go near and separate the dogs when you see them fighting \|  \|  \| \| 1. Cover your face/head with *Gho or Tego* and scroll down to the ground if a dog started biting you \|  \|  \| \| 1. Runaway fast if a dog started biting you \|  \|  \| \| 1. Call the pack of dogs and give your leftover food (lunch) to the dogs \|  \|  \| \| 1. Wash the hands after toughing or playing with the dogs \|  \|  \| \| 1. When dog is angry, they show their teeth, growl and pull their tail straight up in the air \|  \|  \| \| 1. Dog should be approached slowly and confidently, let them sniff your hand and pet them on back before touching \|  \|  \| \| 1. Dog bite in the face is more dangerous than bite on the leg \|  \|  \| | |

**Thank you for your participation!**
